# Malarial Hemoglobinuria*Read before meeting of the East Texas Medico-Chirurgical Society at Palestine, Texas, May 31, 1901.

**Published:** 1901-12

**Authors:** J. H. Paxton

**Affiliations:** Elkhart, Texas


For Texas Medical Journal.

### Malarial Hemoglobinuria.*

BY J. H. PAXTON, M. D., ELKHART, TEXAS.

I use this term in speaking of this disease in preference to malarial hematuria, as I think it a more scientific term. The term malarial hematuria is misleading to students and young practitioners. The microscope has proven time and again that the disease under consideration is not a hematuria, there being no red cells found in the urine, but the color of the urine is due to large amounts of free hemoglobin. I practiced medicine in Houston county, Texas, near the Trinity river, for six or eight years, and during that time I saw quite a number of cases of so-called malarial hematuria, and I had been taught that it was a hematuria. My preceptor had taught me that quinine must not be given in malarial hematuria. The text-books recommended the administration of quinine and ergot and other remedies to control the hemorrhage. I treated these cases according to my teaching. Not one is left to tell the tale. Every case died. Through the researches of Dr. J. W. Roop, of Mist, Ark., I was led to believe that the condition was one in which there was rapid destruction of the red blood corpuscles, one in which the integrity of the red cell was completely destroyed, and the hemoglobin set free. Dr. Roop, in an article published in the December number of the *Therapeutic Gazette,* 1895, recommended the use of quinine hypodermatically in large doses, as much as one hundred and twenty grains in twenty-four hours. Acting on his suggestion, I began the use of quinine hypodermatically, with most happy results.

I regard hemoglobinuria as one of the pernicious forms of malaria, following chronic malarial intoxication. The patient will generally give a history of repeated attacks of some of the different forms of malaria.

In malarial hemoglobinuria we have to deal with a disease that is at once rapid in its course, and if not treated properly is surely fatal in its termination.

---

*Read before meeting of the East Texas Medico-Chirurgical Society at Palestine, Texas, May 31, 1901.

Before outlining my treatment I will mention some of the symptoms and complications that go to make up a clinical picture that once seen is never forgotten.

The patient is generally taken with a severe chill, or he may have had a chill and fever for two days in succession, followed by hyperpyrexia, 103° to 105° F., and voiding of a large amount of dark wine-colored urine, intense discoloration of skin and conjunctiva, which rapidly deepens until patient assumes an olive, and in some cases a mahogony appearance, with a greenish tint. The more rapid the discoloration and the deeper the hue, the graver the prognosis. There is always present intense nausea and vomiting. Vomited matter generally greenish and sometimes of a coffee-ground appearance. Sometimes patient will have rigors every few hours, and these rigors are generally followed by the voiding of a large amount of hemoglobinous urine. Pain in back and loins shooting down into thighs. Intense restlessness; patient rolling from side to side of bed; sometimes suppression of urine, and if this last condition is not relieved, patient gets into a comatose condition and dies. Sometimes acute hepatitis and splenitis supervene, and I have always found it impossible to relieve the patient until this complication is controlled. When patient recovers we have a condition of anemia and weakness to combat, which is slow and tedious. If the patient dies he either dies of asthenia or uremic coma.

*Treatment.*—I begin with hypodermics of morphia and atropia to relieve pain, sick stomach and restlessness, followed by hypodermics of bisulphate of quinine, until I get at least thirty grains in the patient, and I keep up the administration of quinine hypodermically every two to four hours until I give from seventy-five to one hundred and twenty grains in twenty-four hours. I give calomel in five to twenty grain doses every two hours until I have given at least one hundred grains. Then I begin using large enemas of water, sometimes salt and water. I administer hyposulphite of soda in one drachm doses every four hours until I get a thorough evacuation of the alimentary canal, and I keep up the administration of hyposulphite of soda for several days to keep the bowels and liver acting.

For the kidneys I give the mildest diuretics I know of—cit. pot., F. ext. cornsilk, spts. nitre. For restlessness I have found a combination of chloral and bromides with ext. Indian hemp to be very efficacious, especially so if alternated with hypodermics of morph.

I make a routine practice of fortifying the nervous system with strychnine in doses of one-twentieth to one-thirtieth grain every four hours. Mustard plasters over epigastrium and small of back. Iced champagne and cracked ice are gratefully received by the nauseated stomach. Cold water for the hyperpyrexia.

In this disease I believe the coal tar derivatives can do great harm, as they all, to a greater or lesser extent, interfere with oxygen carrying power of the blood, and here we already have the integrity of blood interfered with.

*After-treatment.*—I use tonics of iron, quinine and strychnine, Gude's Pepto Mangan, solution of iron with strychnine and arseniated.

This disease might be more easily handled if we would fortify the patient's blood by administration of normal saline solution by hypodermatoclysis. This measure I have never tried. I treated ten cases last year by the above method, with only one death, and that was a man sixty-five years old.

